# Weight loss intervention for individuals with high internal disinhibition: design of the Acceptance Based Behavioral Intervention (ABBI) randomized controlled trial

**DOI:** 10.1186/s40359-015-0075-2

**Published:** 2015-05-28

**Authors:** Jason Lillis, Heather M Niemeier, Kathryn M Ross, J Graham Thomas, Tricia Leahey, Jessica Unick, Kathleen E Kendra, Rena R Wing

**Affiliations:** The Miriam Hospital, Brown Medical School, Providence, USA; University of Wisconsin, Whitewater, USA; University of Connecticut, Mansfield, USA; Weight Control and Diabetes Research Center, The Miriam Hospital, Brown Medical School, 196 Richmond Street, Providence, RI 02903 USA

**Keywords:** Obesity, Weight loss, Disinhibition, Acceptance, Mindfulness, Emotional eating, Acceptance and commitment therapy

## Abstract

**Background:**

Obesity is public health problem associated with significant health risks and healthcare costs. Behavioral weight control programs produce clinically meaningful weight losses, however outcomes have high variability and maintenance continues to be a problem. The current study is an NIH-funded randomized clinical trial testing a novel approach, Acceptance-Based Behavioral Intervention (ABBI), that combines techniques from standard behavioral treatment (SBT) and Acceptance and Commitment Therapy (ACT). We test this approach among individuals reporting high internal disinhibition who typically respond poorly to standard interventions and appear to benefit from ACT components.

**Methods/Design:**

The ABBI study targets recruitment of 160 overweight or obese adults (BMI of 25–50) who report that they overeat in response to negative emotional states. These individuals are randomly assigned to either (1) ABBI or (2) SBT. Both interventions involve weekly meetings for 22 sessions, bi-weekly for 6 sessions, and then monthly for 3 sessions and both receive the same calorie intake target (1200–1800, depending on starting weight), exercise goal (work up to 250 min per week), and self-monitoring skills training. SBT incorporates current best practice interventions for addressing problematic thoughts and emotions, sometimes called “change” or “control” strategies. ABBI uses acceptance-based techniques based on ACT. Full assessments occur at baseline, 6, 12, and 18 months. Weight loss from baseline to 18 months is the primary outcome.

**Discussion:**

The ABBI study is unique in its focus on integrating acceptance-based techniques into a SBT intervention and targeting a group of individuals with problems with emotional overeating who might experience particular benefit from this novel approach.

**Trial Registration:**

ClinicalTrials.gov, NCT01461421 (registered October 25, 2011)

## Background

### Rationale

Overweight and obesity are significant public health problems in the United States affecting nearly 70 % of American adults (Ogden et al. 2014). Obesity-related medical conditions including coronary heart disease, Type 2 diabetes, degenerative joint disease, and hypertension, are estimated to cost $147 billion per year (Finkelstein et al. 2009), and are associated with the death of an estimated 365,000 Americans each year (Finkelstein et al. 2004).

Behavioral weight loss programs are recommended as the treatment of choice for overweight and obese individuals. Current behavioral weight loss programs consistently produce weight losses of about 8 kg at 6-months and significant health improvements (MacLean et al. 2015). However, despite ongoing treatment contact, many patients achieve their maximum weight loss by 6 months and then gradually regain weight over the remainder of the program (Loveman et al. 2011). In addition, there is considerable variability in outcomes, with some patients achieving much better weight losses than others (MacLean et al. 2015). Novel approaches to the behavioral treatment of obesity are needed to address these limitations.

In recent years, obesity researchers have focused on improving the diet and exercise components of behavioral weight loss programs and studied topics such as the dose of exercise needed or the macronutrient composition of the diet (Wing 2004; Wadden et al. 1988; Jakicic et al. 1999; Murphy et al. 1982). There has been much less attention to the approaches used to deal with emotional overeating. In fact, current behavioral weight loss treatment programs include only 2–3 sessions introducing cognitive restructuring and providing psycho-education regarding emotional eating and stress management (Diabetes Prevention Program Research Group 2002). The approach taken in most existing programs is to teach participants to “control” or “change” their negative thoughts and emotions through distraction, thought stopping, and refocusing strategies.

Recent studies suggest that such control strategies may actually make it more difficult for obese individuals to cope with food cravings and lead to greater consumption of craved foods (Forman et al. 2007a; Hoffman et al. 2009). The adverse effect of “control” strategies is particularly apparent in those who report a high susceptibility to food cues (Forman et al. 2007b).

A new generation of cognitive-behavioral techniques (“Third Wave”), that includes Acceptance and Commitment Therapy [ACT; (Hayes et al. 1999)], has shifted the focus from changing internal thoughts and feelings to accepting thoughts and feelings to thereby promote engaging in behavior that is consistent with personal values and life goals (Hayes et al. 2006; Ost 2008). In ACT, the emphasis is placed on increasing awareness and engaging in valued behavior even when unwanted thoughts and feelings are present. Increasing acceptance and reducing excessive attempts to change or control thoughts and feelings has been shown to predict reductions in binge eating, (Telch et al. 2001) alcohol abuse, (Brown et al. 1997) and smoking (Brown et al. 2002).

Several recent studies have evaluated the benefits of incorporating ACT approaches into weight control programs. In an initial study evaluating ACT for weight maintenance, 84 overweight individuals who had lost weight within the past 2 years were randomly assigned to a wait list control group or a 1 day mindfulness and acceptance-based workshop targeting obesity-related stigma and psychological distress (Lillis et al. 2009). The primary outcome was weight maintenance over the subsequent three months; Participants in the ACT group lost 1.6 % of their body weight over the three month follow-up, whereas the control group gained .3 % (medium effect; d = .63). Changes in acceptance of negative thoughts and feelings were each shown to mediate the effect of the intervention on weight loss outcomes (Gifford & Lillis 2009; Lillis et al. 2009).

Forman and colleagues conducted a 12-week open trial, which served as the first test of a combined standard behavioral + ACT intervention (Forman et al. 2009). Results showed 4.5 % weight loss post-treatment and 6.6 % at 6-month follow-up for intent-to-treat, and 6.6 % and 9.6 % respectively for completers (64 %). Based on these positive outcomes, the authors then conducted a randomized trial comparing the combined treatment (referred to as acceptance-based behavioral treatment, or ABT) to SBT (Forman et al. 2014). Both groups produced significant weight loss and the overall weight losses did not differ between groups. However a post-hoc analysis suggested that when administered by experts, weight loss was significantly higher in ABT than SBT at post-treatment (13.2 % v. 7.5 %) and 6-month follow-up (10.9 % vs. 4.8 %). In addition, the ABT approach was found to be particularly effective in participants who reported high levels of emotional eating and disinhibition at post treatment (12.6 % vs 8.2 % and 12.3 % vs. 10.4 % respectively) and 6-month follow-up (10.5 % vs 6.0 %; 8.3 % vs. 6.3 %).

Internal disinhibition, or the tendency to overeat or lose control of eating in response to negative cognitive or emotional cues is typically assessed using the disinhibition subscale of the Eating Inventory (Stunkard & Messick 1985). This subscale includes two factors: internal disinhibition which is the tendency to eat in response to negative cognitive or emotional cues, and external disinhibition which is the tendency to eat in response to environmental cues (Niemeier et al. 2007). In recent studies, external disinhibiton did not predict weight loss outcomes, but higher baseline levels of internal disinhibition (Niemeier et al. 2007) and a smaller decrease in internal disinhibiton early in weight loss treatment (Butryn et al. 2009) predicted poorer weight loss outcomes. Since ACT emphasizes acceptance of negative thoughts and emotions, rather than trying to change or control them, programs which incorporate ACT components may be particularly effective with this subgroup.

Niemeier and colleagues (Niemeier et al. 2012) conducted an uncontrolled pilot study of a combined SBT + ACT intervention (referred to as ABBI) with 21 overweight or obese men and women who were selected based on their self-reported tendency to experience internal disinhibition. Participants lost an average of 12.0 kg after 6 months of treatment and maintained that weight loss during an untreated follow-up period of three months. Additionally, greater decreases in avoidance of weight-related negative thoughts and feelings were associated with greater weight loss. Both these weight losses, which compare favorably with the standard weight loss literature, and the results from the trial by Forman et al. suggest that ACT approaches may be particularly effective for this subgroup. However, to date, there has never been a trial comparing a weight loss program based solely on standard behavioral strategies with a program that combines SBT plus ACT in the treatment of overweight or obese participants who report high internal disinhibition.

#### Specific Aims

The primary aim of this study is to conduct a randomized controlled trial comparing standard behavioral weight loss treatment (SBT) with a program which combines standard behavioral weight loss components with acceptance-based strategies from ACT (which we have called Acceptance Based Behavioral Intervention or ABBI) in the treatment of overweight and obese individuals who report high internal disinhibition. We proposed a total of 160 participants who were overweight or obese and scored high on internal disinhibition.

The primary hypothesis is that participants in the ABBI program will achieve better weight losses at 6, 12, and 18 months than participants in SBT.

Secondary hypotheses are: (1) Participants in ABBI will experience greater improvements in acceptance of weight related negative thoughts and emotions and distress tolerance at 3 and 9 months than participants in SBT. (2) If the primary and secondary hypothesis #1 are confirmed, we will examine the extent to which the temporally precedent changes in acceptance of weight related negative thoughts and emotions and distress tolerance mediate subsequent differences in weight loss between the two groups.

Additional measures are included to examine the impact of the interventions on weight-related behaviors (diet and exercise) and psychosocial outcomes.

## Method

### Study Design

The current study is a randomized controlled trial. Primary eligibility criteria are having a BMI between 25 and 50 and reporting high internal disinhibition. Potential participants are screened on the phone and must attend an orientation session and a baseline assessment appointment before being randomized to one of the two treatment groups: SBT or ABBI. Both interventions involve face-to-face group meetings weekly for 6 months, bi-weekly for 3 months, and then once per month for the final 3 months. Full assessments occur at baseline, 6, 12, and 18 months. In addition, mediators are measures at 3 and 9 months.

### Research Site

All study activities take place at the Weight Control and Diabetes Research Center (WCDRC) in Rhode Island, United States. The WCDRC is a joint research institution of The Miriam Hospital and the Brown University Medical School.

### Inclusion Criteria

Inclusion criteria are 18–70 years of age, BMI between 25–50 kg/m^2^, and a score of 5 or higher on the internal disinhibition (ID) subscale of the Eating Inventory. Previous research has shown that individuals who score 5 or higher (out of 8) on the ID subscale lose significantly less weight in a standard behavioral weight loss program over 18 months [4.8 kg vs. 7.6 kg; 27]. (Table 1)

**Table 1:** List of internal disinhibition scale questions

Eating Inventory Question (number)
(9) When I feel anxious, I find myself eating.
(11) Since my weight goes up and down, I have gone on reducing diets more than once.
(20) When I feel blue, I often overeat.
(27) When I feel lonely, I console myself by eating.
(36) While on a diet, if I eat a food that is not allowed, I often then splurge and eat other high calorie foods.
(45) Do you eat sensibly in front of others and splurge alone?
(49) Do you go on eating binges even though you are not hungry?
(50) To what extent does this statement describe your eating behavior? “I start dieting in the morning, but because of any number of things that happen during the day, by evening I have given up and eat what I want, promising myself to start dieting again tomorrow.”

### Exclusion Criteria

Participants are excluded for the following safety and retention related issues: Currently in another weight loss program and/or are taking a weight loss medication or has lost ≥ 5 % of body weight during the past six months; currently pregnant, lactating, less than 6 months post-partum, or plans to become pregnant during the next 18 months; reports a heart condition, chest pain during periods of activity or rest, or loss of consciousness on the Physical Activity Readiness Questionnaire (Thomas et al. 1992); reports a medical condition that would affect the safety of participating in unsupervised physical activity; unable to walk 2 blocks without stopping; reports conditions that in the opinion of the investigators would render them potentially unlikely to follow the protocol, including terminal illness, plans to relocate, or a history of substance abuse, bulimia nervosa, or psychiatric hospitalization.

### Recruitment

Participants are recruited through local newspaper advertisements that are designed to target individuals who might score high on internal disinhibition and included phrases such as, “Do you have trouble controlling your eating when you are stressed?” and, “Would you consider yourself an emotional eater?” In addition, to recruit a more diverse sample, direct mailing are used. Recruitment materials with pictures of men and people from a variety of racial and ethnic backgrounds and using the term “stress eating” (rather than emotional eater) are sent to zip-codes with higher representation of minorities.

### Enrollment Procedure

#### Phone Screen

Participants make the initial contact via telephone in response to advertisements or direct mailings and are briefly screened to determine initial eligibility based on the criteria listed above. If deemed potentially eligible, participants are invited to attend an orientation session.

#### Orientation, Run-in Period and Baseline Assessment

**Fig. 1:**
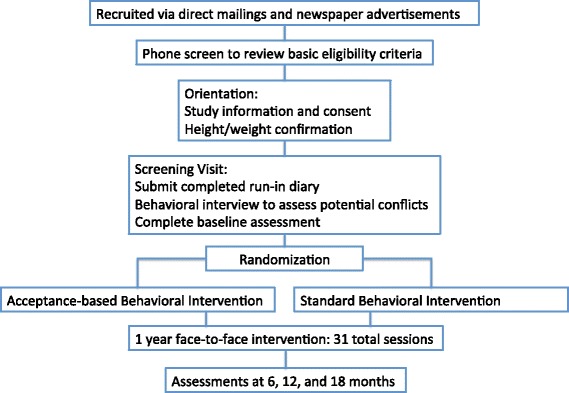
Study design

The orientation session provides detailed information about study procedures and those who are interested in participating signed an IRB approved consent form. Subsequently participants are asked to keep a detailed food diary for one week (serving as a run-in period) and then to attend a baseline assessment, where they are interviewed to assess for potential barriers to completing the program (e.g., extended travel plans, lack of transportation, etc.…). Eligible participants who attend the baseline assessment, complete the run-in diary, and indicate no major barriers to attending sessions are then randomized and allocated to treatment. Randomization is simple 1:1 allocation using number generating software. However, given the expected low number of males, randomization is separated by gender to ensure near equal numbers of males and females in each condition. (Fig. 1)

### Outcome Measures

Research staff members who are blinded to participants’ treatment assignment administer all assessments. The full set of measures is collected at baseline, 6, 12 and 18 months; body weight and the proposed mediators are also assessed at 3 and 9 months so that these variables can be examined prospectively as predictors of subsequent changes in outcomes.

#### Anthropometric

The primary outcome is weight change. Weight is measured to the nearest 0.1 kg using a digital scale and height is measured to the nearest millimeter with a stadiometer, using standardized procedures. Participants are measured wearing light indoor clothing without shoes. BMI will be calculated by formula (kg/m^2^).

#### Diet, Exercise, and Eating Behavior

##### Paffenbarger Physical Activity Questionnaire

This self-report measure of physical activity assesses blocks walked, stairs climbed, and sports activities over the prior week. The data provide a measure of caloric expenditure in overall activity and in light, moderate, and high intensity activities. Changes on the Paffenbarger have been shown to relate to weight loss and weight regain in a large number of behavioral studies (Pronk & Wing 1994; Jakicic et al. 2008).

##### Block Food Frequency Questionnaire

The Block Food Frequency questionnaire (Block et al. 1990) asks participants to indicate how often they have consumed specific foods and their average portion sizes and provides information about total calories and percent of calories from fat, protein, and carbohydrates. This measure has been used in DPP, Look AHEAD, and in other behavioral weight loss studies and changes in percent of calories from fat have been correlated with weight change (Jeffery et al. 1993; McGuire et al. 1999).

##### Eating Inventory

The Eating Inventory (EI) is a widely used measure of eating behavior that includes three subscales, cognitive restraint, disinhibition, and hunger (Stunkard & Messick 1985). The disinhibition scale will be divided into two subscales, internal and external disinhibition. The EI has demonstrated adequate internal consistency and test-retest reliability. Changes in all three subscales have been seen in many prior weight loss studies (Wing & Phelan 2002; Wing et al. 2008). Higher scores indicate more of a given variable.

##### Weight Control Strategies Scale

The WCSS is a 30-item self-report measure used to assess the use of specific strategies for losing or maintaining weight loss (Pinto et al. 2013). The WCSS contains 4 subscales: Dietary Choices, Self-monitoring Strategies, Physical Activity, and Psychological Coping. Higher scores indicate greater use of weight control strategies. The WCSS has been show to have good reliability and validity for use in overweight and obese weight loss treatment seeking samples (Pinto et al. 2013).

#### Psychosocial Measures

##### PROMIS Initiative Short-Forms

Depression, anxiety, quality of life, and satisfaction with relationships were assessed using standardized measures from the NIH PROMIS (Patient Reported Outcomes Measurement Information System) initiative (DeWalt et al. 2007). The Depression-Short Form measures depression using 4 self-report, likert scale items. Higher scores indicate more depression. The Anxiety-Short Form measures anxiety using 4 self-report, likert scale items. Higher scores indicate more anxiety. The PROMIS Global form is a 10-item self-report measure that assess physical and mental quality of life. Higher scores indicate better quality of life. The Satisfaction with Relationships-Short Form measures relationship satisfaction using 4 self-report likert, scale items. Higher scores indicate greater satisfaction with relationships. PROMIS measures are well-established with population norms and good validity (DeWalt et al. 2007).

##### Eating Disorder Examination-Questionnaire

The EDE-Q is a self-report version of the interviewer based eating disorder examination. The Binge Eating subscales (6 items) were used in this study to assess binge episodes occurring within the last 28 days that are both unusually large and associated with a loss of control. The use of laxatives and vomiting as a means of controlling weight are also assessed.

##### Bull’s Eye

The Bull’s Eye (Lundgren et al. 2012) assesses the ability to take action consistent with one’s stated values and goals. Participants identify their personal values and goals in four areas (health, relationships, work, leisure) and then indicate on a dartboard how consistent their behavior has been with those stated values and goals, with marks closer to the center indicating greater consistency. Marks are converted into a Likert scale from 1–7, with higher scores indicating greater consistency of behavior to stated values. The Bull’s Eye has shown good reliability and validity (Lundgren et al. 2012).

#### Theoretical Mediators

##### Acceptance and Action Questionnaire-Weight

The AAQ-W is a 22-item questionnaire that assesses experiential avoidance related to body weight, food and eating. Higher scores indicate more weight-related experiential avoidance. The AAQ-W has demonstrated good reliability and validity and has been show to mediate outcomes in ACT interventions for weight control (Lillis & Hayes 2008; Lillis et al. 2009).

##### Acceptance and Action Questionnaire-II

The acceptance and action questionnaire II (AAQ) is a seven-item questionnaire that assesses general experiential avoidance (Bond et al. 2011). Higher scores indicate more experiential avoidance. The AAQ has good reliability and validity and is associated with a wide range of psychosocial and behavioral health outcomes (Bond et al. 2011).

##### Avoidance and Inflexibility Scale

The AIS is a 13-item questionnaire that assesses avoidance and inflexibility in the face of thoughts, feelings, and bodily sensations. Higher scores indicate greater levels of avoidance and inflexibility. The AIS was used initially with smoking cessation (Gifford et al. 2004), but has been modified to be appropriate for weight control. Gifford and Lillis (Gifford & Lillis 2009) reported that changes on the AIS mediated the effect of ACT on change in BMI.

##### Breath Holding

We use breath holding as an objective measures of distress tolerance because it has been shown to relate to outcomes in a variety of areas (Brown et al. 2002; Brown et al. 2009), and was shown previously to mediate the effects of an ACT-based treatment on weight control (Lillis et al. 2009). In the breath holding (Hajek et al. 1987) task, participants are asked to breathe normally for 30-s, exhale on cue, and then take a deep breath and hold it for as long as possible. Time elapsed is measured by a stopwatch. Two trials are completed and the trial of the longest duration is used. (Table 2)

**Table 2:** Assessment schedule

Study Month	0	3	6	9	12	18
Baseline Questionnaire (Demographics)	X					
Weight	X	X	X	X	X	X
Paffenbarger	X		X		X	X
Block Food Frequency	X		X		X	X
Eating Inventory	X		X		X	X
Weight Control Strategies Scale	X		X		X	X
PROMIS Short forms	X		X		X	X
Eating Disorder Examination-Q	X		X		X	X
Bull’s Eye	X		X		X	X
Acceptance and Action Questionnaire-W	X	X	X	X	X	X
Acceptance and Action Questionnaire-II	X	X	X	X	X	X
Avoidance and Inflexibility Scale	X	X	X	X	X	X
Breath Holding	X		X		X	X

### Interventions

The intervention is delivered in group format with 15–16 participants per group. Groups meet weekly during months 1–6, then bi-weekly during months 6–9, and then monthly during months 9–12 for a total of 31 sessions. Groups are scheduled for 1 h. Group leaders conduct a brief check-in and weigh participants prior to each session. There is no treatment contact between month 12 and the final assessment at month 18. (Table 3)

**Table 3:** Schedule of intervention contact

Time Frame	Frequency	Total
Months 1-6	Weekly	22
Months 7-9	Bi-weekly	6
Months 10-12	Monthly	3

The groups are run by co-leader pairs, which include a mix of Ph.D. psychologists, Ph.D. exercise physiologists, and master’s level nutritionists. Each leader pair is responsible for running both conditions in the cohort in order to counterbalance leader effects. All the group leaders have training and experience running standard behavioral weight loss interventions. Experience with acceptance-based interventions varied from novice (newly trained for the current study) to expert. All group leaders received a 2-day training in acceptance-based interventions and meet for weekly supervision with one of the study co-investigators.

All sessions are audiotaped for treatment fidelity analysis.

#### Shared Components

Both intervention conditions share core components that make up gold standard behavioral weight loss treatment.

##### Weight loss goals

Participants are encouraged to lose 1 to 2 lb per week and to achieve and then maintain a weight loss of 10 % of initial body weight.(Look AHEAD Research Group 2006)

##### Diet

Participants are placed on a standard calorie and fat restricted diet, with goals of 1200–1800 kcal/day and 33–42 g of fat/day (25 % calories from fat) depending on their baseline weight. This approach is typically used in behavioral weight loss programs and is consistent with AHA and ADA guidelines. (Look AHEAD Research Group 2006) Sample meal plans are provided and participants are given a fat/calorie guidebook and instructed to self-monitor their daily calorie and fat intake in their food diaries. Diaries are reviewed each week by the interventionists who provide written feedback to participants.

##### Exercise

Participants are encouraged to gradually increase their physical activity until they are exercising at least 250 min per week at moderate intensity (goal = 50-75 % of maximal heart rate, not to exceed perceived exertion of 13 on a 6–20 scale); typically by using brisk walking or another desired activity.

##### Behavior Therapy

Participants are taught standard behavioral strategies to assist in the modification of their eating and exercise habits including self-monitoring (Baker & Kirschenbaum 1993; Boutelle & Kirschenbaum 1998), stimulus control, problem-solving (Perri et al. 2001), assertiveness training, social support (Wing & Jeffery 1999), goal setting (Bandura & Simon 1977), and relapse prevention (Marlatt & Gordon 1985). For individuals who reach the weight loss goal, maintenance is emphasized. Later lessons include relapse prevention, dealing with motivation erosion, improving the quality of the diet through approaches such as volumetrics, and adding novelty to the physical activity regimen.

#### Components that differ in ABBI vs SBT

The SBT intervention addresses negative thoughts and emotions in three sessions during the first 22 weeks and reviews core skills during the reduced contact phases. To address thoughts that may impede weight loss, participants are taught to recognize a negative thought, stop it, and replace it with a positive thought. Different types of negative thoughts (rationalizations, dichotomous thinking, etc.) are described and participants practice positive ways of reframing them. To reduce stress and change eating in response to emotions, relaxation techniques are presented and distraction and increased participation in pleasurable (non-eating) activities are encouraged. This approach is sometimes described as “change-focused” because modifying negative thoughts and emotions is assumed to thereby change associated maladaptive behaviors.

In contrast, the ABBI intervention teaches acceptance, mindfulness, and values-based techniques to address negative thoughts, emotions, and food cravings (Hayes et al. 2012; Lillis et al. 2014). These techniques are taught individually (each of 3 components is taught in 2 sessions for a total of 6 sessions) and then integrated into the treatment overall. Experiential methods are utilized, where participants are presented key metaphors and engage in activities designed to illustrate key points.

Acceptance strategies are introduced by demonstrating through experiential exercises that efforts to control or avoid internal experiences have not been successful and are actually linked with unsuccessful weight control behaviors. For example, emotional eating is discussed as a way to reduce stress or sadness in the short-term, at the expense of more stress and sadness, reduced health, and possibly increased weight over the medium to long-term. Efforts to control unwanted feelings right now can often create more negative feelings and behavioral outcomes later. This is referred to as the cost of avoidance, or non-acceptance, of emotions. Alternatively, mindful acceptance is taught in relation to unwanted emotions and food cravings. A variety of exercises are used to expose participants to unwanted physiological and emotional states (through guided imagery and the presentation of desired foods), and then distress tolerance skills, such as urge surfing, are taught in vivo with unwanted emotions or cravings present.

Mindfulness techniques help participants increase awareness of their thoughts and feelings. One particular form of mindfulness emphasized in ABBI is cognitive defusion, which aims to help participants distance themselves from unhelpful thoughts without trying to change or get rid of them. The primary goal of defusion work is to de-couple problematic thoughts from unhealthy behavior. Participants are taught many strategies that include increased awareness of thoughts through meditation, thought labeling (e.g., “self-sabotaging” or “judgment”), guided imagery (e.g., imaging thoughts as leaves on a stream), thought exposure (repeating a problematic thought over and over), and metaphor (e.g., imagining your mind as a “bad motivational speaker”).

Values work helps participants identify how weight-related behaviors fit with their core values. In this context, weight influencing behaviors are seen as supporting a broader picture of desired life actions that includes possibly being active, nourishing your body, setting a good example for family members, and increasing longevity to spend more time with loved ones. Goals support values by providing tangible markers along the way, such as losing 10 lb or exercising 5 times this week, however the goals are not presumed to have any meaning or importance outside the context of stated values. The connection of weight-influencing behaviors to core values is repeatedly emphasized and presumed to sustain motivation to persist over time.

### Treatment Fidelity

Detailed patient and counselor manuals are used for all group sessions and all treatment staff are required to carefully read and review these manuals prior to session. Weekly supervision sessions are conducted with current interventionists and led by a co-investigator. In addition, all treatment sessions are audio-taped and a random set of 20 % are coded based on a standardized treatment fidelity rating form that was designed to (1) assure that core treatment elements were presented, and, (2) detect contamination of distinct intervention methods (e.g., acceptance strategies being used in the SBT condition).

### Sample Size Considerations

The primary outcome of the current study is weight loss over the 18 months in ABBI versus SBT, As we expect the variability in weight changes between individuals to be the largest at the 18-month assessment (and thus, result in larger standard deviations around mean weight losses at that time point), we conducted our power analyses to detect differences in weight loss between ABBI and SBT at Month 18. Power analyses were completed using linear mixed effects models (similar to the proposed main model) on 10,000 simulated datasets (simulations based on data from our previous pilot work). The proposed sample of 160 (n per group of 80) has 89 % power for the primary model to detect differences over time of 2.4 kg at 6 months, 3.0 kg at 12 months, and 3.6 kg at 18 months (the power for follow-up analyses detecting between-group differences each individual time point was found to be 93 % at 6 months, 85 % at 12 months, and 83 % at 18 months). The mediation analysis specified in the secondary hypothesis is exploratory in nature, and thus was not included in power analysis considerations.

### Analysis and Statistical Methods

#### Missing data

Following a documented pattern of weight regain following the cessation of treatment (Jeffery et al. 2000; Wadden et al. 2013), missing data are assumed to be missing not at random (MNAR). Thus, we use sensitivity analyses based on multiple-imputation models (Rubin 1987) to explore how robust our findings are with respect to a range of assumptions regarding missing data.

#### Primary Aim

The primary aim of the current study, examining differences in weight loss between the SBT and ABBI groups across the 18-month trial, will be investigated using a longitudinal mixed effects model. Conditionally upon finding a significant omnibus test (at α = 0.05), we will examine between-group differences at 6, 12, and 18 months.

#### Secondary Aim

The secondary aim, testing whether participants in the ABBI group experience greater improvements in acceptance of weight-related negative thoughts and distress tolerance at 6, 12, and 18 months compared to SBT participants, will be tested using a similar model to that described in the primary aim. We will further assess whether participants in the ABBI group, compared to participants in the SBT group, have better adherence to the program (assessed by session attendance) and larger changes in caloric intake/physical activity through use of generalized linear mixed models. Finally, if these between-group differences are confirmed, we will examine the extent to which changes in acceptance variables mediate differences in weight loss between the two study arms. Specifically, we will determine whether changes in acceptance from baseline to 3, 9, and 12 months mediate differences in weight loss between the ABBI and SBT groups at 6, 12, and 18 months, respectively, using a multivariate mediation model.

### Assessment of Safety

The current protocol is approved by The Miriam Hospital Institutional Review Board (TMH IRB). The potential risks to participants in the current trial are considered to be minimal. The intervention recommends a weight loss of 1–2 lb per week and a diet that is balanced (with caloric intakes of 1200 to 1800 kcal/day, based on baseline weight). The physical activity recommendation is for moderate-intensity activities with only gradual increases in the amount of physical activity.

A detailed safety monitoring plan, including oversight from two external safety officers experienced with large weight management trials, has been created for the current study. Tables indicating progress with recruitment, retention at assessment sessions, reasons for dropping-out, and adverse events are submitted to safety officers annually for review. Adverse events are reported continuously to TMH IRB, and if deemed necessary the study sponsor.

### Data management, protection and confidentiality

Every effort is made to maintain confidentiality of all study participants. During the initial phone screen, potential participants are given a unique identification number (with no references to an individual’s name, address, or phone number) that is used on all documents. All data is stored in locked filing cabinets in locked rooms, or electronically on computers with secure passwords. A separate file linking study ID and participant identifiers (e.g., name, address, phone number, and contact names and addresses) is maintained in a password protected electronic file.

## Summary

The ABBI study is a randomized controlled trial comparing standard behavioral treatment (SBT) to an acceptance-based treatment (ABBI) for the purpose of improving 18-month weight loss among adults who report high internal disinhibition. The ABBI study is unique in its focus on integrating acceptance-based techniques into a SBT intervention and targeting a group of individuals with problems with emotional overeating.
